# MRI radiomics-based interpretable model and nomogram for preoperative prediction of Ki-67 expression status in primary central nervous system lymphoma

**DOI:** 10.3389/fmed.2024.1345162

**Published:** 2024-06-27

**Authors:** Endong Zhao, Yun-Feng Yang, Miaomiao Bai, Hao Zhang, Yuan-Yuan Yang, Xuelin Song, Shiyun Lou, Yunxuan Yu, Chao Yang

**Affiliations:** ^1^Department of Radiology, The First Affiliated Hospital of Dalian Medical University, Dalian, Liaoning, China; ^2^Laboratory for Medical Imaging Informatics, Shanghai Institute of Technical Physics, Chinese Academy of Sciences, Shanghai, China; ^3^Laboratory for Medical Imaging Informatics, University of Chinese Academy of Sciences, Beijing, China; ^4^Department of Radiology, The Second Affiliated Hospital of Dalian Medical University, Dalian, Liaoning, China

**Keywords:** Ki-67, radiomics, interpretable model, primary central nervous system lymphoma, nomogram

## Abstract

**Objectives:**

To investigate the value of interpretable machine learning model and nomogram based on clinical factors, MRI imaging features, and radiomic features to predict Ki-67 expression in primary central nervous system lymphomas (PCNSL).

**Materials and methods:**

MRI images and clinical information of 92 PCNSL patients were retrospectively collected, which were divided into 53 cases in the training set and 39 cases in the external validation set according to different medical centers. A 3D brain tumor segmentation model was trained based on nnU-NetV2, and two prediction models, interpretable Random Forest (RF) incorporating the SHapley Additive exPlanations (SHAP) method and nomogram based on multivariate logistic regression, were proposed for the task of Ki-67 expression status prediction.

**Results:**

The mean dice Similarity Coefficient (DSC) score of the 3D segmentation model on the validation set was 0.85. On the Ki-67 expression prediction task, the AUC of the interpretable RF model on the validation set was 0.84 (95% CI:0.81, 0.86; *p* < 0.001), which was a 3% improvement compared to the AUC of the nomogram. The Delong test showed that the z statistic for the difference between the two models was 1.901, corresponding to a *p* value of 0.057. In addition, SHAP analysis showed that the Rad-Score made a significant contribution to the model decision.

**Conclusion:**

In this study, we developed a 3D brain tumor segmentation model and used an interpretable machine learning model and nomogram for preoperative prediction of Ki-67 expression status in PCNSL patients, which improved the prediction of this medical task.

**Clinical relevance statement:**

Ki-67 represents the degree of active cell proliferation and is an important prognostic parameter associated with clinical outcomes. Non-invasive and accurate prediction of Ki-67 expression level preoperatively plays an important role in targeting treatment selection and patient stratification management for PCNSL thereby improving prognosis.

## Highlights

- This is the first study to utilize radiomics to preoperatively predict Ki-67 expression status in primary central nervous system lymphoma;
- An interpretable machine learning algorithm framework is proposed to bridge the performance and interpretability gap of traditional classification algorithms;
- A 3D automated brain tumor segmentation model was developed to provide a convenient automated segmentation tool for subsequent brain tumor-related studies.

## Introduction

PCNSL is a rare malignant tumor that involves only the Central nervous system (CNS) without lymphomas occurring elsewhere. PCNSL accounts for 3% of CNS tumors and more than 90% are diffuse large B cell lymphomas (1). The incidence of this disease has increased exponentially over the past few decades (2).

The Ki-67 proliferation index has been used as a surrogate marker for rapid growth and increased invasiveness in tumors, and an increasing number of researchers have attempted to predict its expression status in a variety of tumors by different methods, Ki-67 has emerged as one of the major predictive factors for tumor prognosis (3–13). Multiple studies have revealed the prognostic significance of Ki-67 in PCNSL, demonstrating significant independent predictive value (14–21). Liu et al.’s research indicates that high Ki-67 expression (i.e., Ki-67 index ≥90%) is associated with poorer overall survival and progression-free survival in PCNSL (18, 22, 23). Currently, the conventional methods for detecting Ki-67 expression status are utilizing biopsy or surgery, but the risk of intracranial complications is high. Therefore, accurate preoperative noninvasive prediction of Ki-67 expression levels plays an important role in targeting therapeutic choices and patient management for PCNSL thereby improving prognosis.

Accurate semantic segmentation of medical images can help doctors pinpoint pathological areas and help in disease research (24, 25). nnU-NetV2 is a deep learning-based semantic segmentation method, which, as the most competitive medical image segmentation model, achieves optimal results in most public semantic segmentation tasks (26). Radiomics allows high-throughput extraction of key features of an image and utilizes these features in combination with machine learning algorithms to make predictions. Studies have shown that most complex and efficient machine learning models lack interpretability (27, 28). SHAP (29) is a method that provides an explanation for the model by calculating SHAP values to quantify the impact of each feature on the predicted results. In addition, multimodal information also adds to model predictions (30).

Therefore, this paper proposes an interpretable machine learning model that incorporates multimodal information such as clinical factors, image features, and radiomics features to compensate for the shortcomings of existing models in terms of performance or interpretability. In addition, the Nomogram method, which is commonly used in clinical research, is constructed for model comparison experiments. To achieve the goal of effectively predicting the Ki-67 expression status in PCNSL before surgery.

## Materials and methods

### Patients

This study was approved by the ethics committees of Medical Center 1 and Medical Center 2. The informed consent of the patient is waived, and the entire study follows the principles outlined in the Declaration of Helsinki.

**Figure 1:**
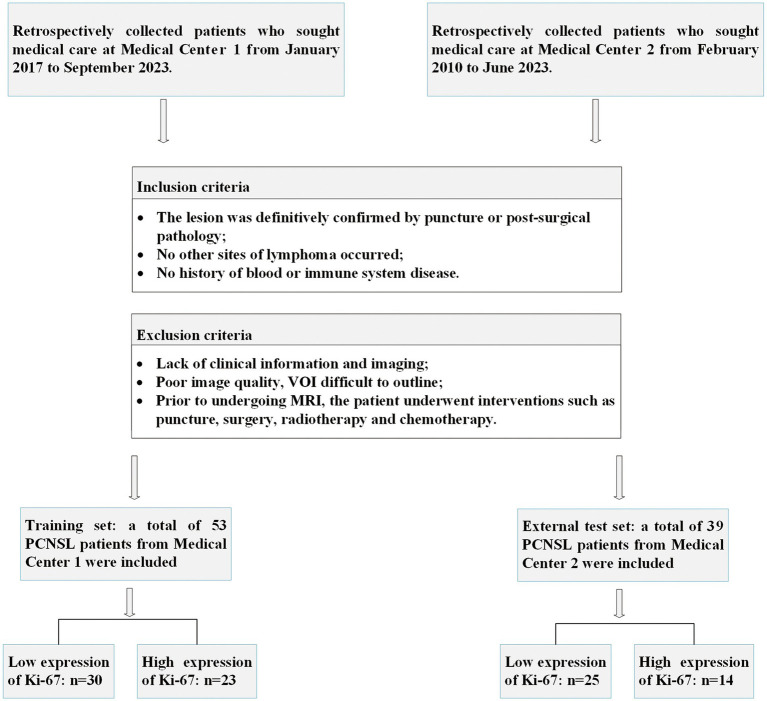
Patient enrollment flowchart.

Patients with primary central nervous system lymphoma attending Medical Center 1 from January 2017 to September 2023 and Medical Center 2 from February 2010 to June 2023 were retrospectively collected. Inclusion criteria: (1) The lesion was definitively confirmed by puncture or post-surgical pathology; (2) No other sites of lymphoma occurred; (3) No history of blood or immune system disease. Exclusion criteria: (1) Lack of clinical information and imaging; (2) Poor image quality, VOI difficult to outline; (3) Prior to undergoing MRI, the patient underwent interventions such as puncture, surgery, radiotherapy, and chemotherapy. A total of 92 patients were finally enrolled in the study and the pathological types are all diffuse large B-cell lymphoma, including 53 in the training set (Medical Center 1) and 39 in the external validation set (Medical Center 2). The enrollment flow chart is shown in Figure 1.

### Imaging protocol

The following MRI scanners were used: GE Signa HDXT 1.5 T 、Siemens Verio 3.0 T and Philips Ingenia CX 3.0 T. More detailed scanning parameters can be found in the Supplementary Appendix.

### Image pre-processing

Patient information was first anonymized for all image sequences and then the original DICOM images of three different sequences (T1WI, T1CE, and T2WI) were converted to Neuroimaging Informatics Technology Initiative (NIFTI) format. The spatial resolution of all image sequences is resampled to [1 mm, 1 mm, 3 mm] by the nearest-neighbor interpolation algorithm, which ensures the consistency of the physical space in different images and removes part of the bias caused by different instruments. The MRI images were corrected for artifacts using the N4 bias field correction algorithm. Finally, the Min-max method was used to normalize the intensity of all images into the 0–255 interval. Preprocessing of all images was done using the Simple ITK algorithm library (https://simpleitk.org/).

### Imaging features analysis

Analysis of the patient’s clinical information, MRI imaging features, and histopathology data. Includes analysis of patients’ clinical information, MRI image characteristics, and histopathologic data (31, 32). Included: (1) Age and Sex; (2) Tumor length: the maximum diameter of the tumor was measured on the image of the largest cross-section of the tumor; (3) Edema volume: the edema VOI volume parameter was calculated by the 3D Slicer’s calculation function; (4) Involvement of deep regions: whether the tumor invades periventricular regions, basal ganglia, brainstem, or cerebellum; (5) Cystic and necrosis: yes, no; (6) Tumor margins: regular, irregular; (7) Enhancement features: mass and patchy, indicates obvious solid enhancement without large areas of non-enhancing necrosis within. Ring enhancement refers to a circular ring of peripheral enhancement due to cystic degeneration and necrosis within the tumor, leading to no enhancement in the interior. (8) Enhanced signal: homogeneous, nonhomogeneous; (9) Midline shift: yes, no; (10) Morphological characteristics: Angular sign, the irregular enhancement lesions protrude to a certain direction, showing a sharp angle appearance; Incision sign, based on the T1CE images, there are umbilical concave or striated defects on the edge of the enhanced lesion; Butterfly sign, lesion involving the corpus callosum can infiltrate transcallosally, appearing as a symmetric “butterfly” appearance n T1CE imaging; (11) Ki-67index: the Ki-67 proliferation index was calculated using the percentage of cells staining positive for Ki-67.

Imaging features were evaluated by 2 radiologists with 3 years of experience in diagnostic CNS imaging. When disagreements in assessment arose, they were resolved by another radiologist with 15 years of experience in diagnostic CNS imaging. All radiologists were blinded to the patient’s histopathologic information when evaluating imaging features.

### Immunohistochemical

Surgical specimens were fixed in 10% buffered formalin solution and then wax block embedded, sectioned, and stained with anti-Ki67 antibody. The antibody binds to the Ki67 protein and the positive cells show a brown complex. One thousand cells were randomly selected in the hot spot field for Ki-67-positive cell counting, and the percentage of all counted cells was the Ki-67 index. According to previous studies (18, 22, 23), 90% was used as the cutoff value for the Ki-67 index. Ki-67 index ≥90% was defined as high expression and less than 90% indicated low expression. There were 37 cases in the high-expression group and 55 cases in the low-expression group.

### Research analysis workflow

**Figure 2:**
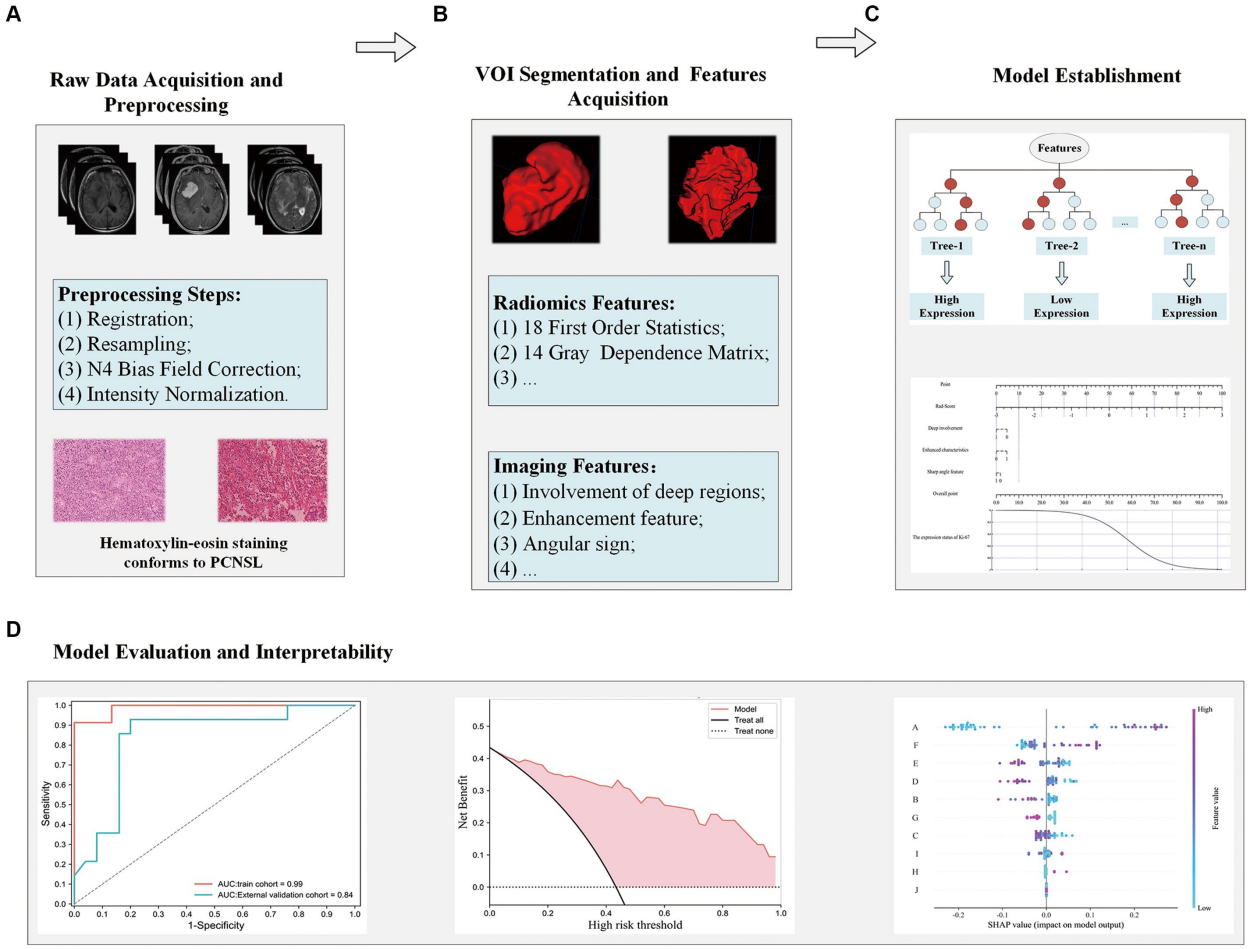
Flowchart for analyzing radiomics modeling studies. Raw data acquisition and preprocessing **(A)**; VOI segmentation and features acquisition **(B)**; Model establishment **(C)**; Evaluation of the model and interpretability analysis **(D)**.

The research pipeline of this paper is mainly divided into the following four modules: A. Raw Data Acquisition and Preprocessing; B. VOI Segmentation and Features Acquisition; C. Model Establishment; D. Evaluation of the model and interpretability analysis. The specific information is shown in Figure 2.

### The volume of interest segmentation and image registration

**Figure 3:**
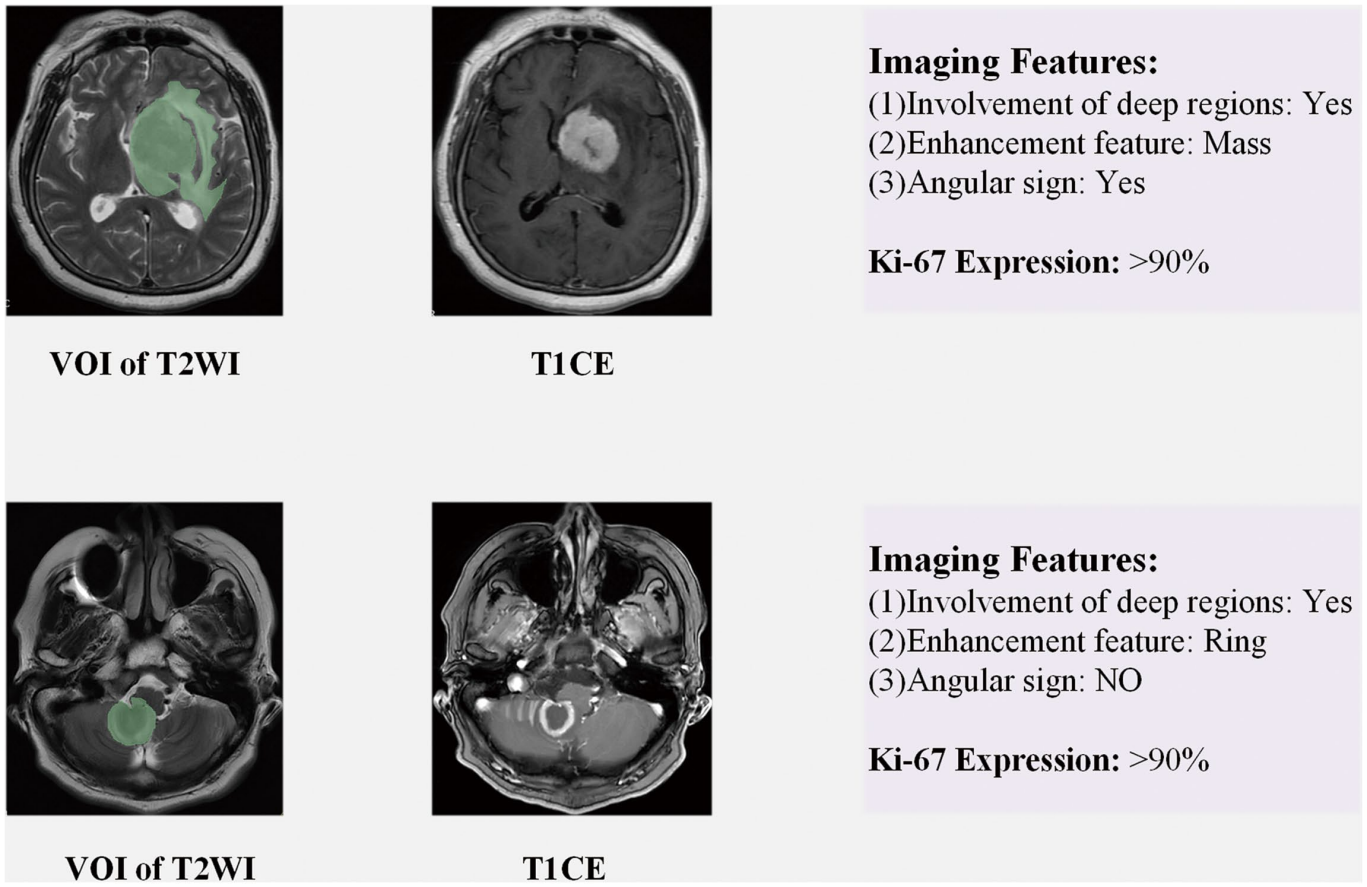
Schematic diagram of VOI segmentation and partial image features. The case in the first line depicts a tumor in the left basal ganglia region, which exhibits three imaging features (Involvement of deep regions, Angular sign, Enhancement features: Mass); The case in the second row shows tumors in the cerebellum, including Involvement of deep regions, Enhancement features (Ring), and no Angular sign. Involvement of deep regions: whether the tumor invades periventricular regions, basal ganglia, brainstem, or cerebellum. Angular sign:the irregular enhancement lesions protrude to a certain direction, showing a sharp angle appearance. Enhancement features (Mass): the tumor shows obvious massive. Enhancement features (Ring): there is no obvious enhancement inside the tumor, but obvious circular enhancement can be seen at the edges.

The preprocessed T2WI sequences were imported into 3Dslicer (version 5.0.2) software, and VOI segmentation of the tumor parenchyma and its peritumoral edema region was performed by a radiologist with 3 years of experience. The final obtained VOI is used as Ground Truth for training the 3D automatic segmentation model. From all images, twenty T2WI sequences were randomly taken and segmented by another radiologist with 3 years of experience to prepare the data for ICC calculation. The results of the segmentation are shown schematically in Figure 3. The first case depicts a tumor located in the left basal ganglia region, affecting deep brain tissue, with angular protrusions visible at the front edge and an overall nodular enhancement. The second case shows a tumor in the cerebellum, also within deep brain tissue, with a smooth tumor edge and a ring-shaped enhancement on the contrast-enhanced scan.

The 3D automatic segmentation model developed in this study is trained based on nnU-NetV2. nnU-NetV2 is an improved version of the U-Net model. U-Net is a classic convolutional neural network architecture for image segmentation, and nnU-Net builds upon it with enhancements, particularly excelling in medical image segmentation tasks. For detailed technical details about the nnU-NetV2 model, please refer to the Supplementary Appendix. The experiments were configured with Python 3.9.0, Pytorch 2.0.0, and cuda11.8 deep learning platforms, Windows 10 operating system, and NVIDIA GeForce RTX 4090 GPU. An efficient 3D segmentation model for brain tumors was obtained by using the 3d_fullres mode for the training setup of the network, and the pre-processed 3D MRI images and Ground Truth were inputted into the network for training. The model is based on a large number of public dataset training experiences to summarize some of the fixed hyperparameters and configuration experience, and can automatically configure the hyperparameters of the model for any new dataset’s characteristics (including Learning rate, Loss function, Architecture template, Optimizer, Data augmentation, etc.), to learn from the *a priori* experience of other medical datasets while avoiding the problem of model performance degradation due to the lack of experience (5). The final training and validation results of the model are evaluated using the DSC metric.

For image alignment, the Simple ITK algorithm library and ITK-Snap software (version: 4.0.1, www.itksnap.org) were used to align the images of T1WI and T1CE to T2WI. Mutual information was used as the Image similarity metric, Rigid was used as the Transformation model, and Nearest Neighbor Interpolation was used as the image interpolation method.

### Feature extraction and screening

Based on Ground Truth and 3 MRI sequences (aligned T1WI sequence, aligned T1WI-enhanced sequence, T2WI sequence), using the Pyradiomics (https://pyradiomics.readthedocs.io/en/latest/) algorithm library (33) to extract 1762 radiomics features, all of which were compliant with the Image Biomarker Standardization Initiative (IBSI) standard (34). Radiomics features obtained from the three sets of sequences were combined to obtain a total of 5,286 features, and the intraclass correlation coefficient (ICC) was calculated based on duplicate outlined image sequences of 20 cases to remove unstable features (ICC < 0.85). Then *Z*-Score normalization was performed and redundant features were removed using Pearson or Spearman methods. Finally, the radiomics features were screened using the minimal-redundancy-maximal-relevance criterion (mRMR) feature screening algorithm. To further improve the prediction performance of the model, the fusion feature Rad-Score is computed using ElasticNet and added to the training process of the model. For detailed information on the mRMR method, the extracted radiomics feature categories and Rad Score, please refer to the Supplementary Appendix.

Univariate logistic regression analysis was performed on the clinical information and MRI image features, and the variables with *p* < 0.05 were selected for the multivariable logistic regression analysis, and finally the variables with *p* < 0.05 were selected as independent predictors of Ki-67 expression status and were used to construct the interpretable machine-learning model and nomogram.

### Radiomics model construction

The construction steps of interpretable machine learning predictive models can be divided into three main parts: extraction and screening of structured features, training of complex machine learning models, and application of model interpretation methods. The screened multimodal factors such as multisequence radiomics features and coded clinically independent predictors were first combined and screened using the mRMR algorithm to obtain the 10 predictors with the most predictive value. The Synthetic Minority Oversampling Technique (SMOTE) (35) technique is used to balance the number of samples across labels in the training set to improve the upper bound of model performance. The Random Forest algorithm was chosen as the classifier for model training, and 5-fold cross-validation was used in the training process. The optimal parameter search was performed using GridSearchCV to obtain the best model. The SHAP method was used to provide an explanation for the prediction results of the final random forest model. The principles and advantages of SHAP model interpretable methods and SMOTE data augmentation methods can be found in the Supplementary Appendix.

In addition, independent predictors and the Rad-Score fusion feature screened by univariate and multivariable analyses were combined, and the multivariable logistic regression model was used for model training and the construction of a nomogram.

### Statistics

SPSS 26.0 (https://www.ibm.com/spss) and Python 3.7 (https://www.python.org/) were used for statistical analysis. Measurements were tested for normality, and data that did not fit the normal distribution were expressed as median (interquartile spacing) M (P25, P75). Comparisons between groups were made by the Mann–Whitney U rank sum test. The chi-square test or Fisher’s exact probability method was used for counting data. Results of logistic regression analyses were expressed as odd ratio (OR) and 95% CI. *p* < 0.05 represents a statistical difference. Use “sklearn. Metrics,” “sklearn. Calibration,” and “matplotlib. Pyplot “to calculate the AUC, Sensitivity, and Specificity of the model and plot the calibration curve and decision curve of the model, respectively. The Delong test was used to compare the AUC of the models.

## Results

### Patient characteristics

Table 1 provides detailed results of the differential analysis of clinical factors and imaging characteristics. It can be found that the distribution of patient information was balanced between the training cohort and the external test cohort, with no statistically significant difference in any of the distributions (*p* > 0.05). In addition, Table 2 shows that the involvement of deep regions, enhancement feature, and angular sign were statistically significant (*p* < 0.05) in the Ki-67 high and low expression groups.

**Table 1:** Results of the variability analysis of clinical factors and imaging features between the training set and the external test set.

Clinical information and MRI features	Total (*n* = 92)	Training cohort (*n* = 53)	External validation cohort (*n* = 39)	*P*
Age, M (P25, P75)	62.00 (57.25–67.75)	63.00 (58.00–68.00)	62.00 (55.00–67.00)	0.420
Tumor length (mm), M (P25, P75)	33.50 (26.00–47.50)	32.00 (22.50–43.00)	36.00 (30.00–48.00)	0.140
Edema volume (cm^3^), M (P25, P75)	51.67 (21.32–91.37)	42.67 (20.72–94.62)	55.37 (26.36–84.44)	0.890
Sex, *n* (%)				0.217
Male	47 (51.09)	30 (56.60)	17 (43.59)	
Female	45 (48.91)	23 (43.40)	22 (56.41)	
Ki-67, *n* (%)				0.469
Ki-67 < 90%	55 (59.78)	30 (56.60)	25 (64.10)	
Ki-67 ≥ 90%	37 (40.22)	23 (43.40)	14 (35.90)	
Involvement of deep regions, *n* (%)				0.488
Yes	58 (63.04)	35 (66.04)	23 (58.97)	
No	34 (36.96)	18 (33.96)	16 (41.03)	
Cystic and necrosis, *n* (%)				0.650
Yes	21 (22.83)	13 (24.53)	8 (20.51)	
No	71 (77.17)	40 (75.47)	31 (79.49)	
Tumor margin, *n* (%)				0.219
Regular	29 (31.52)	14 (26.42)	15 (38.46)	
Irregular	63 (68.48)	39 (73.58)	24 (61.54)	
Enhancement feature, *n* (%)				1.000
Mass and Patchy	85 (92.39)	49 (92.45)	36 (92.31)	
Ring	7 (7.61)	4 (7.55)	3 (7.69)	
Enhanced signal, *n* (%)				0.236
Homogeneous	56 (60.87)	35 (66.04)	21 (53.85)	
Nonhomogeneous	36 (39.13)	18 (33.96)	18 (46.15)	
Midline shift, *n* (%)				0.235
Yes	50 (54.35)	26 (49.06)	24 (61.54)	
No	42 (45.65)	27 (50.94)	15 (38.46)	
Angular sign, *n* (%)				0.503
Yes	27 (29.35)	17 (32.08)	10 (25.64)	
No	65 (70.65)	36 (67.92)	29 (74.36)	
Incision sign, *n* (%)				0.172
Yes	49 (53.26)	25 (47.17)	24 (61.54)	
No	43 (46.74)	28 (52.83)	15 (38.46)	
Butterfly sign, *n* (%)				0.132
Yes	5 (5.43)	5 (9.43)	0 (0.00)	
No	87 (94.57)	48 (90.57)	39 (100.00)	

**Table 2:** Results of the differential analysis of clinical factors and imaging characteristics between the Ki-67 high and low expression groups.

Clinical information And MRI features	Total (*n* = 92)	Ki-67 < 90% (*n* = 55)	Ki-67 ≥ 90% (*n* = 37)	*P*
Age, M (P25, P75)	62.00 (57.75–67.75)	63.00 (57.00–68.00)	62.00 (57.00–67.00)	0.786
Tumor length (mm), M (P25, P75)	33.50 (26.00–47.50)	34.00 (25.00–50.00)	33.00 (27.00–45.50)	0.802
Edema volume (cm^3^), M (P25, P75)	51.67 (21.32–91.37)	42.43 (20.24–89.18)	64.68 (25.32–92.85)	0.438
Sex, *n* (%)				0.097
Male	47 (51.09)	32 (58.18)	15 (40.54)	
Female	45 (48.91)	23 (41.82)	22 (59.46)	
Involvement of deep regions, *n* (%)				**0.012**
Yes	58 (63.04)	29 (52.73)	29 (78.38)	
No	34 (36.96)	26 (47.27)	8 (21.62)	
Cystic and necrosis, *n* (%)				0.196
Yes	21 (22.83)	10 (18.18)	11 (29.73)	
No	71 (77.17)	45 (81.82)	26 (70.27)	
Tumor margin, *n* (%)				0.762
Regular	29 (31.52)	18 (32.73)	11 (29.73)	
Irregular	63 (68.48)	37 (67.27)	26 (70.27)	
Enhancement feature, *n* (%)				**0.031**
Mass and Patchy	85 (92.39)	54 (98.18)	31 (83.78)	
Ring	7 (7.61)	1 (1.82)	6 (16.22)	
Enhanced signal, *n* (%)				0.272
Homogeneous	56 (60.87)	36 (65.45)	20 (54.05)	
Nonhomogeneous	36 (39.13)	19 (34.55)	17 (45.95)	
Midline shift, *n* (%)				0.217
Yes	50 (54.35)	27 (49.09)	23 (62.16)	
No	42 (45.65)	28 (50.91)	14 (37.84)	
Angular sign, *n* (%)				**0.016**
Yes	27 (29.35)	11 (20.00)	16 (43.24)	
No	65 (70.65)	44 (80.00)	21 (56.76)	
Incision sign, *n* (%)				0.467
Yes	49 (53.26)	31 (56.36)	18 (48.65)	
No	43 (46.74)	24 (43.64)	19 (51.35)	
Butterfly sign, *n* (%)				0.632
Yes	5 (5.43)	4 (7.27)	1 (2.70)	
No	87 (94.57)	51 (92.73)	36 (97.30)	

Statistically significant results are in bold (*p* < 0.05).

### Evaluation of 3D segmentation model based on nnU-NetV2

**Figure 4:**
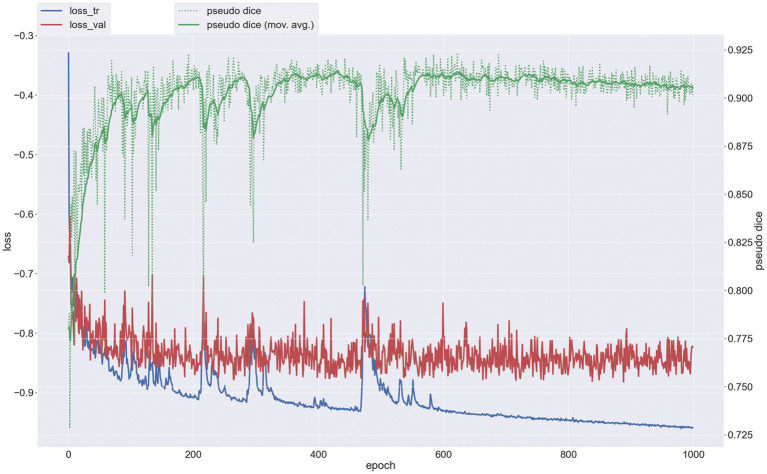
Training iteration plot for 3D brain tumor segmentation model.

The configured Batch size for training the segmentation model is 2 and the Patch size is (48, 224, 192). A total of 1,000 epochs were trained to obtain the optimal model, and the average DSC value on the validation set was 0.85, which shows that nnU-NetV2 has a good segmentation efficacy for brain tumor VOI. The training iterations of the segmentation model are given in Figure 4.

### Performance of the two Ki-67 expression prediction models

A total of 21 unstable radiomics features were removed from all radiomics features by calculating the ICC values, and then 826 features were obtained after de-redundancy using Pearson and Spearman. The fusion feature Rad-Score is computed by the ElasticNet regression network.

**Figure 5:**
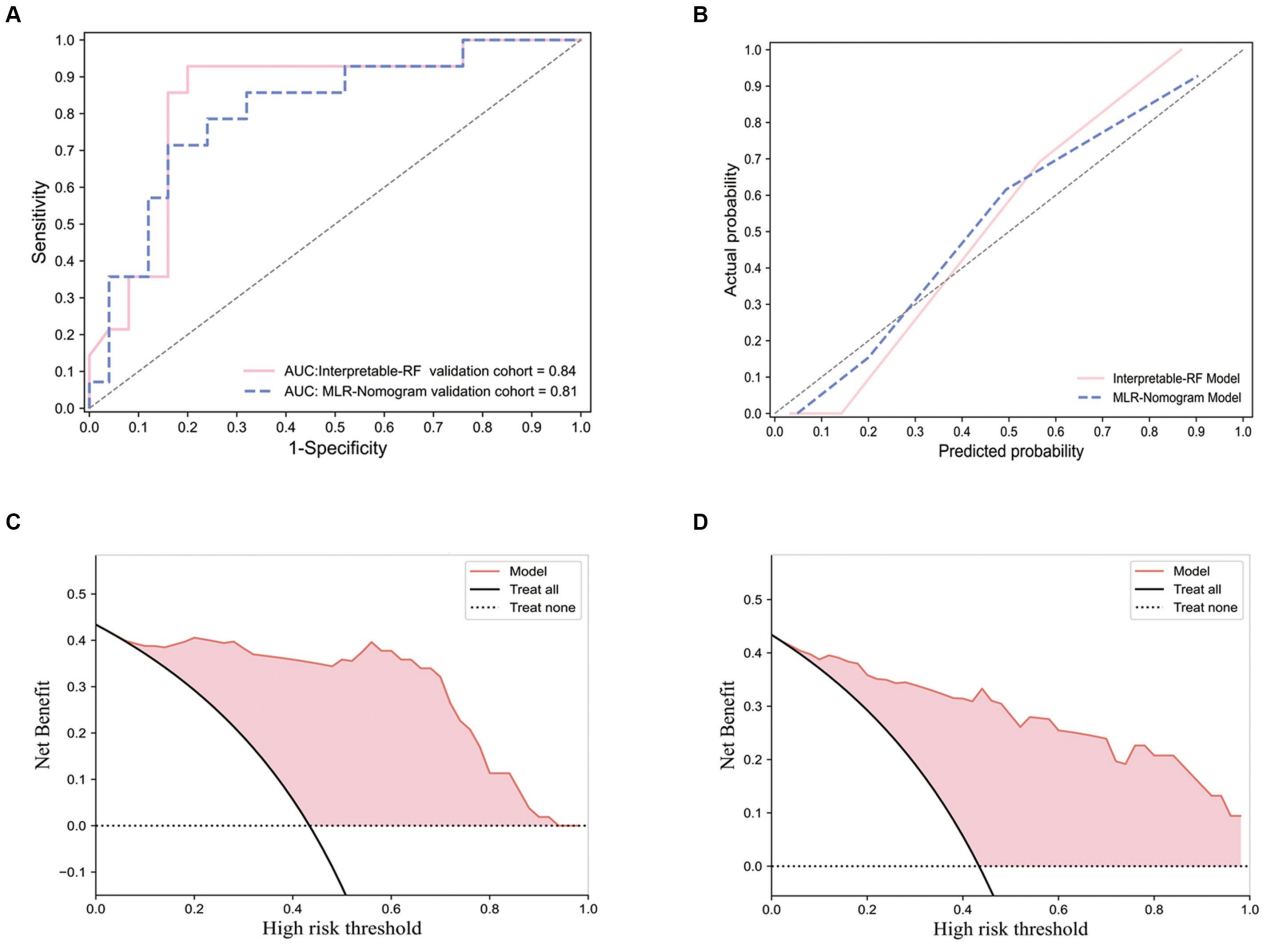
Receiver operating characteristic curve, calibration curves and decision curve analysis curves for the interpretable RF model and the multivariate logistic regression nomogram model. The ROC performance of the interpretable RF model and the multivariate logistic regression nomogram model on an external test set **(A)**; Performance of calibration curves for two models **(B)**; The DCA performance of the interpretable RF model **(C)**; The DCA performance of the multivariate logistic regression nomogram model **(D)**.

For the training of the interpretable RF model, 10 features with optimal predictive value were obtained after combining clinical factors, imaging features, and radiomics features and filtering them using the mRMR algorithm (including Rad-Score, 6 radiomics features, and 3 imaging features). After training, an efficient interpretable RF classification model is obtained with AUC: 0.84 (95% CI, [0.81, 0.86]), Sensitivity: 0.929, and Specificity: 0.68 on the external test set. The ROC of the interpretable RF model on the external test set is shown in Figure 5A. The calibration curve is shown in Figure 5B, which shows that the model is well-calibrated with a good linear fit. Decision curve analysis (DCA) is shown in Figure 5C, which shows that the model has good clinical utility.

**Figure 6:**
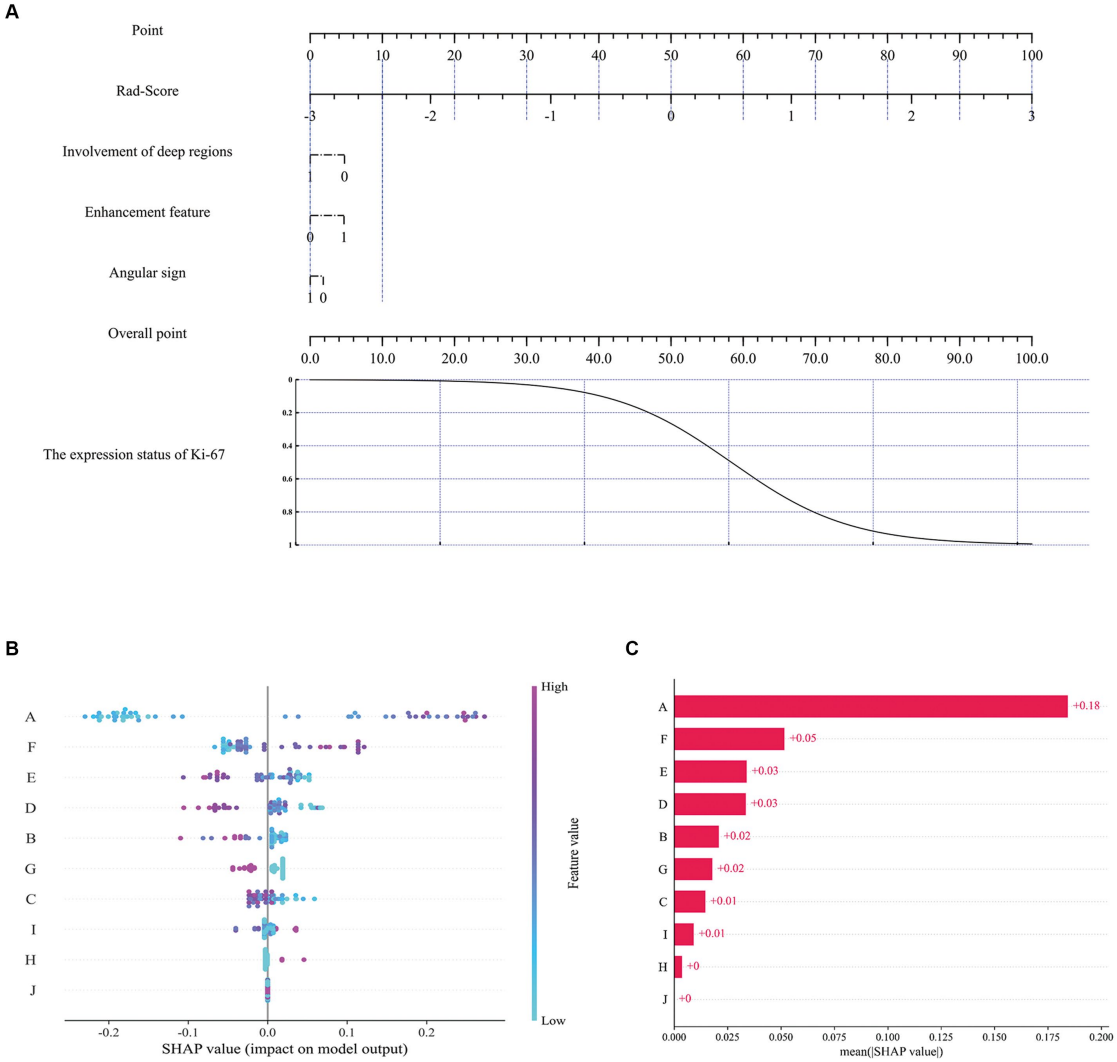
The nomogram and SHAP interpretation plots. Multivariate logistic regression nomogram **(A)**, SHAP model decision interpretation results **(B,C)**. Where A: ‘Rad-score’, B: ‘Gray level co-occurrence matrix correlation’, C: ‘Average gray level intensity’, D:'Gray level dependence matrix dependence entropy’, E: ‘Gray level size zone small area high gray level emphasis’, F: ‘Gray level co-occurrence matrix maximal correlation coefficient’, G: 'Involvement of deep regions’, H: 'Enhancement feature’, I: 'Gray level size zone gray level non-uniformity’, J: 'Angular sign’.

For the multivariate logistic regression nomogram model, three independent predictors associated with Ki-67 expression levels, namely, Deep involvement, Enhanced characteristics, and Sharp angle feature, were screened out from the imaging features and clinical factors using univariate and multivariate analyses, as shown in Table 3. Combining the Rad-Score fusion feature, a nomogram was constructed using a multivariate logistic regression model, as shown in Figure 6A. This model performed in the external test set with AUC:0.81 (95% CI, [0.78, 0.83]), Sensitivity: 0.79, Specificity: 0.72. The Receiver operating characteristic curve (ROC) of this model on the external test set is shown in Figure 5A, which shows a slight decrease in AUC compared to the effect of the interpretable RF model. The calibration curve is shown in Figure 5B, and the DCA is shown in Figure 5D. It can be seen that the model has a good calibration effect and clinical practicability.

**Table 3:** Results of univariate and multivariate analysis of imaging characteristics and clinical factors.

Clinical information And MRI features	Univariate regression analysis		Multivariate regression analysis	
	OR (95%CI)	*P*	OR (95%CI)	*P*
Age, M (P25, P75)	1.01 (0.96–1.06)	0.711		
Tumor length (mm), M (P25, P75)	0.99 (0.96–1.02)	0.566		
Edema volume (cm^3^), M (P25, P75)	1.00 (0.99–1.01)	0.559		
Sex, *n* (%)	2.04 (0.87–4.76)	0.099		
Male				
Female				
Involvement of deep regions	0.31 (0.12–0.79)	**0.014**	0.25 (0.09–0.72)	**0.010**
Yes				
No				
Cystic and necrosis	0.53 (0.20–1.40)	0.199		
Yes				
No				
Tumor margin	1.15 (0.47–2.83)	0.762		
Regular				
Irregular				
Enhancement feature	10.45 (1.20–90.86)	**0.033**	14.51 (1.42–147.89)	**0.024**
Mass and Patchy				
Ring				
Enhanced signal	1.61 (0.69–3.78)	0.273		
Homogeneous				
Nonhomogeneous				
Midline shift	0.59 (0.25–1.37)	0.219		
Yes				
No				
Angular sign	0.33 (0.13–0.83)	**0.018**	0.32 (0.12–0.89)	**0.028**
Yes				
No				
Incision sign	1.36 (0.59–3.15)	0.468		
Yes				
No				
Butterfly sign	2.82 (0.30–26.32)	0.362		
Yes				
No				

Statistically significant results are in bold (*p* < 0.05).

### Feature contribution evaluation by SHAP values

To assess the contribution of each feature to the model, the impact of each feature on the predicted results of the model was quantified using the SHAP method. Previous studies have shown that this method is highly suitable for explaining complex medical artificial intelligence models (36–39). In Figure 6B, each scatter represents a sample, the horizontal axis represents the SHAP values, the vertical axis represents the features, and the position of each feature on the vertical axis indicates its importance, with higher positions indicating larger contributions; the position of the scatter point on the horizontal axis indicates the SHAP value of the feature, the closer the horizontal coordinate is to the centerline (usually zero), the less the feature contributes to the prediction result, and the further the horizontal coordinate is from the centerline, the more the feature contributes to the prediction result; the color of the scatter can be used to indicate the magnitude of the value of the feature. Figure 6C exhibits the SHAP values for different features, and it can be seen that Rad-Score, Gray level co-occurrence matrix maximal correlation coefficient, and Gray level size zone small area high gray level emphasis exhibit higher model contributions, with contribution values of 0.18, 0.05 and 0.03, respectively. For imaging features, the involvement of deep regions showed a relatively high contribution value (0.02).

## Discussion

Ki-67 is a proliferating cell nuclear protein present in all phases of the cell cycle except G0 (40), the higher level indicates more cells in the dividing stage and more active cell division. Ki-67 is an important prognostic parameter associated with clinical outcomes, and accurate preoperative prediction allows for customized patient stratification and proper care. Mukai H’s study suggests that Ki-67 expression levels in patients with breast malignancies may be an indicator of a significant response to neoadjuvant chemotherapy (41). Therefore, the expression level of Ki-67 can also assist in guiding the choice of chemotherapy regimen.

The observation in this study that PCNSL patients in the high-expression group showed more atypical enhancement features, i.e., ring enhancement, was due to the fact that the high Ki-67 index represents excessive tumor growth and high invasiveness, which may lead to cystic degeneration and necrosis due to insufficient blood supply within the tumors; and hemorrhage due to destruction of vascular endothelial cells (42). The “Angular sign” is an intensification of the tumor at a certain level showing a sharp angle of prominence (32), it reflects the characteristics of the lymphoma itself, which has no envelope, is softer in texture, and is easily permeable. This sign appeared more frequently in the Ki-67 high-expression group, which may be due to the uneven growth rate of some parts of the tumor during its rapid growth. In addition, Involvement of deep regions indicates that the tumor growth invades deeper intracranial areas such as periventricular regions, basal ganglia, brainstem, and/or cerebellum [It is an important risk factor in the International Extranodal Lymphoma Study Group (IELSG) prognostic score (43), the present study similarly found that it was also an independent predictor of high Ki-67 expression levels (*p* < 0.05)].

In a previous study, Ouyang et al. demonstrated good discriminatory ability in preoperative prediction of the Ki-67 proliferation index in patients with meningiomas using the radiomics nomogram (44), but they only extracted imaging features of the tumor parenchyma. The tissues surrounding tumors likewise contain a vast amount of heterogeneous information (45), especially vasogenic edema around intracranial tumors, which are sites of altered specific molecular, cellular, biological, and radiological information. Studies in other different classification tasks and our previous studies have shown that the region of the tumor combined with peritumoral edema will effectively improve the diagnostic performance of classification models (46, 47). In this study, peritumoral edema was included in the region of interest together, aiming to maximize the accurate prediction of Ki-67 expression status.

Both the interpretable RF model and the multivariate logistic regression nomogram constructed in this study showed good results in the preoperative prediction of Ki-67 expression status in PCNSL patients. Comparatively, the interpretable RF model showed better predictive performance. Both models use radiomics features and imaging features of multi-sequence MRI images as training data, while the RF model, as a more complex machine learning model, has a higher upper limit of model performance than the logistic regression model, but the RF model’s interpretability is relatively weak, which limits its use for medical tasks (27, 28). This study combines the SHAP method with the RF model to analyze and quantify the impact of each feature on the model’s prediction results, complementing the interpretability of the RF model. In addition, Rad-Score, a more comprehensive and advanced feature, can help the model understand the data better, as can be seen in Figure 6B, where Rad-Score plays an important role in the decision-making process of the interpretable RF model. The SHAP results indicate that among the selected radiomic features, there are three first-order features describing voxel intensity distribution (Gaussian Laplacian operator and wavelet features) and three features quantifying image grayscale (gray-level co-occurrence matrix and gray-level size zone matrix) that are at the forefront. The Gaussian Laplacian operator is a two-dimensional isotropic measure of the image’s second-order spatial derivative, emphasizing regions in the image with rapid intensity changes, thus primarily used in edge detection tasks. Wavelet transform provides a localized analysis of signals in both time and frequency domains, refining the analysis of signals through operations such as dilation and translation at multiple scales, effectively extracting information. Gray-level co-occurrence matrix and gray-level size zone matrix, as texture features in radiomics, primarily describe voxel grayscale distribution and variations, contributing to better prediction of tumor heterogeneity. Additionally, three macroscopic MRI features exhibiting significant differences between enhancement features, involvement of deep regions, and angular sign surpass certain radiomic features, resulting in model benefit. Compared with the multivariate logistic regression nomogram model, this novel interpretable RF model balances the requirements of both model performance and interpretability, improving the prediction level of this task. In terms of automatic brain tumor segmentation, nnU-NetV2 achieves good segmentation performance by implementing automatic network architecture and training hyper-parameter configuration based on the training experience of several public medical databases. Compared to previous studies (10, 11, 13, 48), this study provides a novel interpretable machine learning radiomics framework that offers an efficient solution for the study of other medical tasks.

This study also has some limitations. Two medical center data were included in the study and were validated on an external validation set, but the amount of data from both centers was small. This is mainly because PCNSL is a low-prevalence disease and large-scale data sets are difficult to collect, and data from more medical centers will be included later to increase the data size for further research. In addition, the Ground Truth segmentation labels required for model training at the time of this study still need to be manually labeled by radiologists due to the lack of annotated public datasets relevant to this task. This segmentation work will be a preliminary technology accumulation for later related studies.

## Conclusion

In conclusion, this study presents a novel non-invasive automated interpretable machine learning research framework. The effectiveness of radiomics for preoperative prediction of Ki-67 expression status in PCNSL was explored, and conventional imaging features were incorporated to improve model performance. In addition, an automated 3D brain tumor segmentation model was developed to prepare the segmentation tool for subsequent studies.

## Data availability statement

The original contributions presented in the study are included in the article/supplementary material, further inquiries can be directed to the corresponding author.

## Ethics statement

The studies involving humans were approved by this study was approved by the institutional Ethics Committee of the First Affiliated Hospital of Dalian Medical University (Approval No. PJ-KS-KY-2023-481) and the institutional Ethics Committee of the Second Affiliated Hospital of Dalian Medical University (Approval No. 2023–257). The studies were conducted in accordance with the local legislation and institutional requirements. The ethics committee/institutional review board waived the requirement of written informed consent for participation from the participants or the participants’ legal guardians/next of kin because this study is a retrospective non-interventional study that collected existing radiological data from patients without implementing any additional intervention measures.

## Author contributions

EZ: Conceptualization, Data curation, Formal analysis, Investigation, Methodology, Project administration, Software, Visualization, Writing – original draft. Y-FY: Conceptualization, Data curation, Formal analysis, Investigation, Methodology, Project administration, Software, Visualization, Writing – original draft. MB: Validation, Visualization, Writing – original draft. HZ: Validation, Visualization, Writing – original draft. Y-YY: Supervision, Writing – review & editing. XS: Validation, Visualization, Writing – original draft. SL: Validation, Visualization, Writing – original draft. YY: Validation, Visualization, Writing – original draft. CY: Resources, Supervision, Writing – review & editing.
