# Supplementary material for: MRI radiomics-based interpretable model and nomogram for preoperative prediction of Ki-67 expression status in primary central nervous system lymphoma
Source: Front Med (Lausanne). 2024 Jun 27;11:1345162. doi: 10.3389/fmed.2024.1345162 (PMC11236568; doi:10.3389/fmed.2024.1345162)
Supplement: Supplementary file 1 [file Data_Sheet_1.docx]

**Supplemental material**

**Appendix S1: MRI images acquisition**

Scans were performed using Siemens Verio 3.0T(center 1), GE Signa HDXT 1.5T(center 2), Philips Ingenia CX 3.0T(center 2) MR scanners and their matching head phased array coils.

For GE Signa HDXT 1.5T, T1WI: TR 300ms, TE 9ms; T2WI: TR 4000ms, TE 110ms; Matrix 256*256; Field of View (FOV): 220*220, NEX 2, Slice thickness 6mm, Slice spacing 1mm.

For Siemens Verio 3.0T, T1WI: TR 1750ms, TE 24ms; T2WI: TR 2500ms, TE 120ms; Matrix 320*320; Field of View (FOV): 240*192, NEX 2, Slice thickness 5.5mm, Slice spacing 1.5mm.

For Philips Ingenia CX 3.0T, T1WI: TR 2162ms, TE 15ms; T2WI: TR 2345ms, TE 122ms; Matrix 448*256; Field of View (FOV): 230*230, NEX 2, Slice thickness 6mm, Slice spacing 1mm.

For contrast medium, Gadopentetate Dimeglumine (Gd-DTPA) was injected into the cubital vein using a high-pressure syringe at a dose of 0.1 mmol/kg and a rate of 2.0 mL/s.

**Appendix S2: Radiomics Feature Extraction**

A total of 1762 radiomic features were extracted, which included the following categories: (1) 14 Shape-based features; (2) 18 First Order Statistics features; (3) 23 Gray Level Co-occurrence Matrix features; (4) 16 Gray Level Run Length Matrix features; (5)16 Gray Level Size Zone Matrix features; (6) 5 Neighbouring Gray Tone Difference Matrix features; (7) 14 Gray Level Dependence Matrix features; (8) 736 Wavelet features; (9) 460 Laplacian of Gaussian features; (10) 92 Square features; (11) 92 Square Root features; (12) 92 Logarithm features; (13) 92 Exponential features; (14) 92 Gradient features.

**Appendix S3: Distribution of Ki-67 index in the selected samples**

Table A. Distribution of Ki-67 index in the selected samples.

| **Ki-67 expression** | **90%-100%** | **80%-90%** | **70%-80%** | **60%-70%** | **50%-60%** |
| --- | --- | --- | --- | --- | --- |
| Number | 37 | 33 | 10 | 4 | 8 |

**Appendix S4: The detailed interpretation of the SHAP method and Rad-Score, as well as an explanation of the basic principles and functions of the SMOTE, mRMR, and nnU-NetV2 methods.**

SHAP (SHapley Additive exPlanations)[1] is a method used to explain the predictions of machine learning models. It is based on the concept of Shapley values in game theory, aiming to provide a consistent and mathematically sound explanation for the contribution of each feature. The core idea of SHAP is to decompose the impact of feature values on the model's prediction into contributions from each feature value. In this way, we can understand the importance of each feature for the final prediction. The calculation of SHAP values considers all possible combinations of each feature to determine the relative importance of each feature to the model output. Using SHAP values can help: 1. Understand model predictions by explaining the impact of each feature on the model's predictions to better understand the behavior of the model; 2. Determine feature importance: identifying which features are most critical to the model's predictions; 3. Detect interactions between features, as SHAP values can reveal how interactions between features influence the model's predictions. SHAP is a model-agnostic method for post-hoc explanation, constructing an additive explanation model where all features are considered "contributors." For each predicted sample, the model generates a prediction value, and the SHAP value is the numerical allocation for each feature in that sample. The basic idea is to calculate the marginal contribution when a feature is added to the model, considering the different marginal contributions of this feature in all possible feature sequences, and then taking the average, which becomes the SHAP value for that feature.

The Rad-Score mentioned in this study is obtained by multiplying the selected key features and their corresponding coefficients obtained through ElasticNet and summing them up. It is a comprehensive indicator that integrates multiple imaging omics features into a single numerical value to characterize the biological properties, prognosis, or treatment response of tumors or other diseases. Its main function is to simplify a large number of imaging omics features into a biologically meaningful and clinically applicable index. For example, the Rad-Score can be used to predict the malignancy of tumors, patients' survival period, chemotherapy response, etc. Through the Rad-Score, clinicians and researchers can more easily apply complex radiomics information to clinical decision-making and research.

SMOTE (Synthetic Minority Over-sampling Technique)[2] is a commonly used oversampling method for addressing the issue of imbalanced datasets. In this study, the training set does not maintain a balanced quantity of samples for different labels, therefore, it is necessary to use this method to enhance the training dataset. In imbalanced datasets, the quantity of some classes of samples is significantly less than others, which may cause the model to be biased towards the dominant class during prediction, while overlooking the minority classes. The basic idea of SMOTE is to balance the imbalanced dataset by synthesizing new minority class samples. It achieves this by identifying nearest neighbor samples in the feature space for each minority class sample and inserting new synthetic samples between these neighboring samples to generate new minority class samples. This increases the quantity of minority class samples, making the imbalanced dataset more balanced. The steps of the SMOTE method are as follows: 1. For each minority class sample, identify its k nearest neighbor samples in the feature space (usually using Euclidean distance or other distance measures); 2. Randomly select a sample from these k nearest neighbor samples and randomly generate new synthetic samples along the line segment between this sample and the original sample; 3. Repeat the above steps until the desired quantity of minority class samples is achieved. The synthetic samples generated by the SMOTE method can increase the diversity of minority class samples, thereby improving the model's generalization ability.

mRMR (minimum Redundancy Maximum Relevance)[3] is a feature selection method designed to select the most discriminative subset of features from high-dimensional data. Its core idea is to simultaneously consider the correlation between features and the correlation between features and the target variable, ensuring that the selected features have maximum discrimination and minimum redundancy. The mRMR method is typically implemented by calculating the correlation between features and the target variable, as well as the correlation between pairs of features. Specifically, it first calculates the correlation between each feature and the target variable, and then computes the correlation between pairs of features. Next, it adds the feature with the highest correlation to the already selected feature set, while considering the correlation between this feature and the already selected features to minimize redundancy. This process is repeated until the desired number of features is selected. The mRMR method is widely applied in feature selection, particularly in high-dimensional datasets and fields such as bioinformatics. It helps reduce feature dimensionality, improve the generalization ability of models, and identify the most discriminative features, aiding in the understanding of the structure of the dataset and the relationships between features.

The nnU-NetV2[4] automatic segmentation model is currently one of the most recognized and accurate methods in the medical segmentation field. It has achieved good performance in almost all medical segmentation tasks and is a lightweight deep learning model. nnU-NetV2 is an improved version of the U-Net model. U-Net is a classic convolutional neural network architecture for image segmentation, and nnU-Net builds upon it with enhancements, particularly excelling in medical image segmentation tasks.

The principle of nnU-NetV2 closely follows that of U-Net, consisting of an encoder (down sampling path) and a decoder (up sampling path). The encoder extracts feature from the image and down samples them into a smaller representation, while the decoder maps these features back to the original image size and generates segmentation predictions for each pixel. Compared to traditional U-Net, nnU-NetV2 incorporates several improvements to enhance performance and generalization.

Some advantages of nnU-NetV2 include:

1. Utilization of Data Augmentation Techniques: nnU-Net employs various data augmentation techniques such as random rotation, flipping, scaling, etc., to increase the diversity of training data, thereby improving the model's generalization ability.
2. Model Based on 3D Convolutions: For medical imaging, nnU-Net typically utilizes 3D convolutions to process volumetric data, not just 2D images. This use of 3D convolutions better captures spatial information in volumetric data, enhancing segmentation accuracy.
3. Hierarchical Multiscale Feature Fusion: nnU-NetV2 uses hierarchical multiscale feature fusion mechanism in the decoder to integrate features from different levels of the encoder, better capturing details and contextual information in the images.
4. Adaptive Loss Functions: nnU-NetV2 often employs adaptive loss functions such as Dice loss or cross-entropy loss to better adapt to different types of medical imaging data and segmentation tasks.
5. Utilization of Pretrained Models: Sometimes, nnU-NetV2 may use pretrained models as initial parameters to accelerate the training process and improve the model's performance.

**References**

1 Lundberg SM, Lee S-I (2017) A unified approach to interpreting model predictionsProceedings of the 31st International Conference on Neural Information Processing Systems. Curran Associates Inc., Long Beach, California, USA, pp 4768–4777

2 Chawla NV, Bowyer KW, Hall LO, Kegelmeyer WP (2002) SMOTE: synthetic minority over-sampling technique. J Artif Int Res 16:321–357

3 Ding C, Peng H (2003) Minimum Redundancy Feature Selection From Microarray Gene Expression Data

4 Isensee F, Jaeger PF, Kohl SAA, Petersen J, Maier-Hein KH (2021) nnU-Net: a self-configuring method for deep learning-based biomedical image segmentation. Nat Methods 18:203-211
